# Optimization of Tungsten Heavy Alloy Cutting Parameters Based on RSM and Reinforcement Dung Beetle Algorithm

**DOI:** 10.3390/s23125616

**Published:** 2023-06-15

**Authors:** Xu Zhu, Chao Ni, Guilin Chen, Jiang Guo

**Affiliations:** 1 State Key Laboratory of High-Performance Precision Manufacturing, Dalian University of Technology, Dalian 116024, China; zhuxu@mail.dlut.edu.cn (X.Z.); nichao@mail.dlut.edu.cn (C.N.); cgl@mail.dlut.edu.cn (G.C.); 2 Ningbo Institute of Dalian University of Technology, Ningbo 315000, China

**Keywords:** tungsten heavy alloys, multi-object optimization, dung beetle algorithm, response surface method, multi-sensor

## Abstract

Tungsten heavy alloys (WHAs) are an extremely hard-to-machine material extensively used in demanding applications such as missile liners, aerospace, and optical molds. However, the machining of WHAs remains a challenging task as a result of their high density and elastic stiffness which lead to the deterioration of the machined surface roughness. This paper proposes a brand-new multi-objective dung beetle algorithm. It does not take the cutting parameters (i.e., cutting speed, feed rate, and depth of cut) as the optimization objects but directly optimizes cutting forces and vibration signals monitored using a multi-sensor (i.e., dynamometer and accelerometer). The cutting parameters in the WHA turning process are analyzed through the use of the response surface method (RSM) and the improved dung beetle optimization algorithm. Experimental verification shows that the algorithm has better convergence speed and optimization ability compared with similar algorithms. The optimized forces and vibration are reduced by 9.7% and 46.47%, respectively, and the surface roughness *R*_a_ of the machined surface is reduced by 18.2%. The proposed modeling and optimization algorithms are anticipated to be powerful to provide the basis for the parameter optimization in the cutting of WHAs.

## 1. Introduction

Tungsten heavy alloys (WHAs) possess a unique combination of excellent physicochemical properties, including high density, high mechanical strength, high Young’s modulus, low thermal expansion, good ductility, and excellent resistance to corrosion and radiation, as well as good formability and non-radiation pollution [1,2,3]. They play an irreplaceable role in frontier scientific fields such as rockets, missiles, re-entry spacecraft, and nuclear reactors [4,5,6]. A high-quality machined surface is crucial for the performance of alloy components; therefore, machining is necessary to achieve the desired smoothness/shape and precise dimensions [7]. Due to the fact that WHAs are composite materials prepared via liquid-phase sintering, the presence of tungsten grains, which possess both hardness and brittleness, significantly affects the cutting force and vibration of the cutting tool during the cutting process. Consequently, this leads to the deterioration of the machined surface and reduced machining efficiency.

Currently, research on WHA machining is limited and mainly focuses on areas such as the ultrasonic elliptical vibration cutting and leveraging of single-crystal diamond tools [8,9,10], tool wear mechanisms [11,12,13,14], and chemical–mechanical polishing [15,16]. Yin et al. [8] proposed a design for a single-excitation-based ultrasonic elliptical vibration cutting device. The research results demonstrated that the device performed well in the ultrasonic elliptical vibration cutting of tungsten alloys. Pan et al. applied the ultrasonic elliptical vibration cutting technology to the ultraprecision machining of tungsten alloys, proving that ultrasonic elliptical vibration technology can effectively improve the surface quality of WHAs [9,10]. Further precision processing, such as polishing, of tungsten alloys requires good initial surface conditions of the workpiece. Although ultrasonic elliptical vibration cutting can achieve desirable surface profiles, this machining method imposes specific requirements. Therefore, optimizing the process parameters of the conventional machining of tungsten alloys is necessary to achieve the best possible surface conditions at lower costs.

In recent years, bio-inspired optimization algorithms such as genetic algorithms, artificial bee colony algorithms, and cuckoo search algorithms have achieved significant success in the field of machining [17], which provide novel approaches for optimizing process parameters in machining. Tanvir et al. [18] proposed a hybrid whale optimization algorithm and applied it to optimize the cutting parameters of stainless steel. The results showed significant improvements in the performance of stainless steel turning operations after multi-objective optimization using the hybrid whale algorithm. Vukelic et al. [19] used a genetic algorithm to perform multi-objective optimization of the turning process for 4340 steels. The results demonstrated that optimizing the cutting parameters makes it possible to improve the machining quality while simultaneously reducing the surface roughness and cutting forces. Xue et al. [20] introduced an optimization algorithm inspired by the behavior of dung beetles. This novel algorithm exhibits superior global optimization capabilities compared to other algorithms and has great potential for applications in the field of machining. However, this algorithm is only suitable for handling single-objective optimization problems.

Machining is a non-linear and multi-modal complex process [21]. In the cutting process, besides cutting speed, cutting depth, and feed rate, cutting forces and vibrations also impact the machining quality of the workpiece. Tseng et al. [22] established a cutting force model for low-carbon steel S15C using experimental data and machine learning algorithms, studying the influence of cutting forces on the workpiece surface morphology during turning processes. Segreto et al. [23] used vibration sensors to monitor vibration signals during the turning process of a nickel–titanium alloy. The experimental results showed that parameters such as vibration signal frequency, amplitude, and time-domain features could determine critical indicators such as material removal rate and surface quality during the turning process. When optimizing cutting parameters, it is necessary to consider not only the cutting parameters but also the effects directly caused by forces and vibrations.

In this study, a brand-new multi-objective dung beetle optimization algorithm was proposed to find the optimal processing solution. It introduced the non-dominated sorting technique into the dung beetle optimization algorithm. A regression model for cutting parameters, cutting forces, and vibrations in the machining process of tungsten alloys was established using response surface methodology. The constructed regression model was used as the fitness function to optimize the cutting parameters in the tungsten alloy machining process, obtaining the optimal combination of cutting parameters, which was further validated through experiments. Compared to other multi-objective optimization algorithms, the algorithm proposed in this paper demonstrates a faster convergence speed and superior optimization capability. The following sections will describe the generation of the RSM model, the improvement process of the DBO algorithm, and the optimization process of the cutting parameters.

## 2. Materials and Methods

### 2.1. Workpiece and Cutting Tool

WHA (95W-3.5Ni-1.5Fe) specimens with a diameter of 14 mm were utilized for the experiments. Table 1 illustrates the nominal composition and physical properties of the WHA specimen used in this study. Inserts were composed of cubic boron nitride (CBN) tips brazed onto a WC substrate. The corner radius of the insert was nominally 0.4 mm. These inserts were used with a tool holder with a side rake angle of 35°, a back rake angle of 7°, and a lead angle (DCGW11T304).

### 2.2. Experimental Work

#### 2.2.1. Experimental Setup

**Figure 1:**
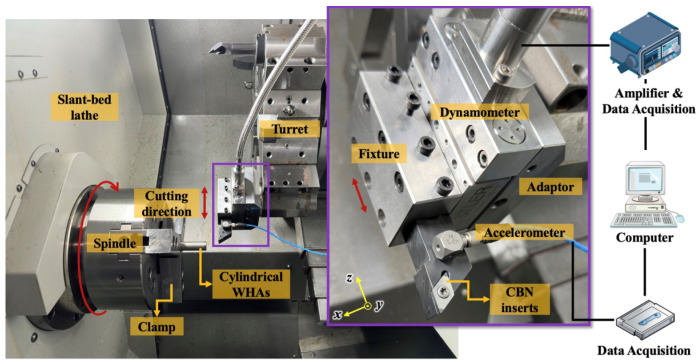
Experimental setup for capturing cutting forces and vibration.

Figure 1 shows the experimental equipment and sensor system used in this study. A cylindrical WHA workpiece in combination was carried out on a slant-bed lathe (Hison, HTC150, Ningbo, China) without cutting fluids. A dynamometer (Kistler, 9119AA2, Winterthur, Switzerland), together with an A/D data acquisition board (Kistler, 5697A, Switzerland), was mounted on the turret of the lathe via an adaptor to capture the cutting forces at a sampling frequency of 5 kHz per channel. The integrated electronic piezo-electric (IEPE) triaxial accelerometer (Kistler, 8763B, Switzerland), connected with data acquisition (National Instruments, Austin, TX, USA), was glued on a tool holder as close to the insert as possible to better measure the vibration generated in the cutting area, with a sampling rate of 5 kHz.

The surface roughness was measured using a stylus profilometer (Mitutoyo, SJ-210, Kawasaki-shi, Japan) and a trace length of 1.6 mm. The surface roughness values were recorded at three equally spaced locations around the circumference of the specimen to obtain statistically significant data for each trial. Inserts were composed of cubic boron nitride (CBN) tips brazed onto a WC substrate. The corner radius of the insert was nominally 0.4 mm. These inserts were used with a tool holder with a side rake angle of 35°, a back rake angle of 7°, and a lead angle (DCGW11T304). In order to reduce the impact of tool wear on the experimental data, a new insert was used for each cutting.

#### 2.2.2. Experimental Design

The response surface method (RSM) is a powerful tool for analyzing the functional relationship between multiple factors with interaction and the response value. It is capable of establishing a high-precision high-order polynomial regression model. Compared to many other predictive models, the response surface method considers the influence of random errors during the experimental process on the model construction. In the field of machining, the response surface method is widely used to analyze the relationship between cutting parameters and material processing characteristics. This study aimed to investigate the influence of each processing parameter on cutting force and vibration. To achieve this, cutting speed, feed rate, and back engagement were selected as variable parameters. Table 2 shows the parameters of the cutting experiments.

A total of 17 experiments were designed using the Box–Behnken design (BBD), and the parameters and results of each experiment are provided in Table 3. It can be seen from Table 3 that the surface roughness of the processed workpiece will decrease with the increase in cutting force and vibration. Therefore, in order to obtain better surface quality, the cutting parameters should be optimized with the optimization goal of reducing cutting force and vibration.

### 2.3. RSM Modeling

RSM is a statistical technique used for modeling any output of interest as a function of contributing independent input variables. The RSM model can be expressed as a polynomial function, typically written in the following format:

(1) $$y=c_{0}+\sum_{i=1}^{n}c_{i}x_{i}+\sum_{i=1}^{n}\sum_{j=1}^{n}c_{\mathrm{ij}}x_{i}x_{j}+\varepsilon$$

wherein ε indicates the fitting error, $c_{0}$ is the regression coefficient of overthinking, and *x_i_* is the input factor of the model. Drawing on research conducted by other scholars in the field of cutting processing, it has been found that the first-order regression prediction model lacks accuracy and adaptability. It is not capable of effectively reflecting the influence of the internal interaction of cutting parameters on the cutting force. Polynomial regression prediction models that go beyond the third order require a significant number of experiments, which will increase the cost and burden of experimentation. Therefore, this study established a quadratic polynomial prediction model for the regression model, which can be expressed as follows:

(2) $$y=c_{0}+\sum_{i=1}^{3}c_{i}x_{i}+\sum_{i=1}^{3}c_{\mathrm{ij}}x_{i}{}^{2}+\sum_{i=1}^{3}\sum_{j=1}^{3}c_{\mathrm{ij}}x_{i}x_{j}+\varepsilon$$

### 2.4. Multi-Objective Optimization Algorithm

#### 2.4.1. Dung Beetle Optimization Algorithm

The dung beetle optimization algorithm is based on the rolling, dancing, breeding, foraging, and stealing behaviors of dung beetles in nature. The population optimization algorithm, the mathematical model of the optimization algorithm, is as follows:

(a) Dung ball

Dung beetles are common insects in nature that feed on animal dung. When the dung beetle rolls the dung ball backward, it can navigate according to the moonlight or sunlight, so that the dung ball moves in a straight line. When there is no light source at all, its trajectory is curved. Assuming that the intensity of the light source will affect the movement path of the dung beetle, its position update in the search space can be expressed as follows:

(3) $$x_{i}\left(t+1\right)=x_{i}\left(t\right)+\alpha \times k\times x_{i}\left(t-1\right)+b\times \Delta x$$

(4) $$\Delta x=\left|x_{i}\left(t\right)-\right.\left.X^{w}\right|$$

where t represents the current iteration number; $x_{i}\left(t\right)$ represents the position of the i’th iteration dung beetle; k∈ (0, 0.2] is a constant value, representing the deflection coefficient; b ∈ (0, 1] is a constant value, a is a natural coefficient, where the value is 1 or −1; $X^{w}$ represents the global worst position; and Δ*x* represents the change in light intensity.

(b) Dance

When dung beetles encounter an obstacle and cannot move, they will dance to the top of the dung ball to reposition themselves and obtain a new route. Assuming that the dung beetle will continue to roll the ball backward immediately after determining the new orientation, the process can be expressed as follows:

(5) $$x_{i}\left(t+1\right)=x_{i}\left(t\right)+\tan\theta \left|x_{i}\left(t\right)-\right.\left.x_{i}\left(t-1\right)\right|$$

wherein θ ∈ [0, π] represents the azimuth angle.

(c) Breed

After the dung beetle transports the dung ball to a safe location and hides it, the female dung beetle will lay eggs in the dung ball. Choosing a suitable place to lay eggs is very important for dung beetles. The region boundary selection strategy for simulating the spawning of dung beetles is defined as follows:

(6) $$Lb^{*}=\max\left(X^{*}\times \left(1-R\right),Lb\right)$$

(7) $$Ub^{*}=max\left(X^{*}\times \left(1-R\right),Ub\right)$$

wherein $X^{*}$ is the current local best position; $Lb^{*}$ and $Ub^{*}$ are the lower bound and upper bound of the spawning area, respectively; *Lb* and *Ub* represent the lower bound and upper bound of the optimization problem, respectively; and R = $1-t/T_{\max}$, $T_{\max}$ represents the maximum number of iterations. Once the spawning area has been determined, the female dung beetle will choose the egg balls in this area to lay eggs. For the DBO algorithm, each female dung beetle lays only one egg in each iteration. The position of the ovum can be expressed as follows:

(8) $$B_{i}\left(t+1\right)=X^{*}+b_{1}\times \left(B_{i}\left(t\right)-Lb^{*}\right)+b_{2}\times \left(B_{i}\left(t\right)-Ub^{*}\right)$$

where $B_{i}\left(t\right)$ is the location information of the i’th egg in the t’th iteration; $b_{1}$ and $b_{2}$ represent two independent random vectors with a size of 1 × D; and D is the dimension of the optimization problem.

(d) Foraging

Some mature small dung beetles will come out of the ground to look for food, and an optimal foraging area needs to be established to guide dung beetles to forage. The boundary of the optimal foraging area is defined as follows:

(9) $$Lb^{b}=max\left(X^{b}\times \left(1-R\right),Lb\right)$$

(10) $$Ub^{b}=max\left(X^{b}\times \left(1-R\right),Ub\right)$$

wherein $Lb^{b}$ and $Ub^{b}$ are the upper bound and lower bound of the optimal foraging area. At this time, the position of the little dung beetle is updated as follows:

(11) $$x_{i}\left(t+1\right)=x_{i}\left(t\right)+C_{1}\times \left(x_{i}\left(t\right)-Lb^{b}\right)+C_{2}\times \left(x_{i}\left(t\right)-Ub^{b}\right)$$

where $C_{1}$ is a random number subject to normal distribution, and $C_{2}$ is a random vector belonging to (0, 1).

(e) Pilfer

There are also some dung beetles called stealing dung beetles, which will steal dung balls from other dung beetles. The DBO algorithm assumes that the stealing behavior occurs at the optimal foraging position $X^{b}$, and the location of the stealing dung beetles is updated as follows:

(12) $$x_{i}\left(t+1\right)=X^{b}+S\times g\times \left(\left|x_{i}\left(t\right)-X^{*}\right|+\left|x_{i}\left(t\right)-X^{b}\right|\right)$$

where g is a random vector with a size of 1 × D, and S is a constant value.

After one iteration, the ball-rolling dung beetle, the brood ball, the small dung beetle, and the thief’s position are updated. The above four agents constitute the population of the optimization algorithm. The DBO algorithm can use the information of different periods to thoroughly explore the search space, avoiding falling into the local optimum, and should have strong searchability.

#### 2.4.2. Multi-Objective Dung Beetle Optimization Algorithm Based on Non-Dominant Ordering

**Figure 2:**
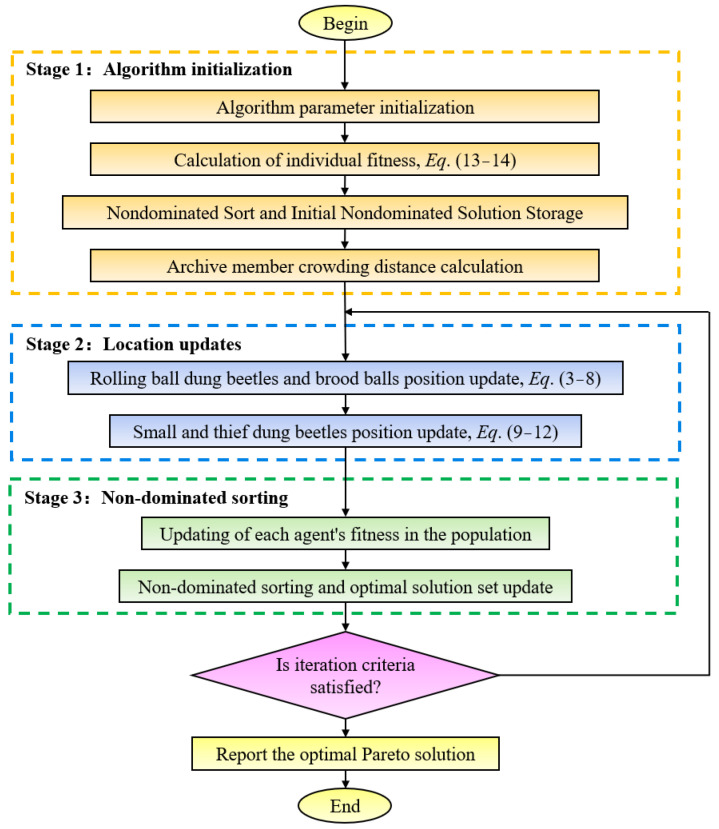
Flowchart of multi-objective dung beetle optimization algorithm.

Non-dominated sorting is one of the most popular and effective techniques in multi-objective optimization algorithms [24]. It sorts and ranks Pareto optimal solutions according to their level of dominance. Among them, solutions that are not dominated by any solution are assigned rank 1. Those dominated by only one solution are assigned rank 2, solutions that are dominated by only two solutions are assigned rank 3, and so on. Afterward, solutions are selected according to their ranks to improve the quality of the population. The flowchart of the NSDBO algorithm is shown in Figure 2.

The optimization process of the NSDBO algorithm mainly includes the following three stages.

Stage 1: Initialize the dung beetle population and algorithm parameters, and assign the proportions of the four agents in the algorithm. Using the constructed $F_{z}$ and $A_{z}$ models as the fitness function, calculate the fitness of each agent in the population. Compute the non-dominated solutions in the initial population and save them in the Pareto archive. Compute the crowding distance for each Pareto archive member.

Stage 2: Update the position of the ball-rolling dung beetle and the brood ball using Formulae (3)–(8). Update the position of the small dung beetle and the thief beetle using Formulae (9)–(12). Eliminate agents that exceed the boundary and generate corresponding new individuals.

Stage 3: Calculate the fitness value of each agent in the population after updating the position. Identify new non-dominated solutions in the population and save them in the Pareto archive, and eliminate the dominant solutions in the Pareto archive. Perform non-dominated sorting and update the Pareto optimal solution. Repeat the above process until the iteration termination condition is satisfied.

## 3. Results and Discussion

### 3.1. Analysis of Experimental Results Based on RSM

In this study, Design-Expert 13 software was used as an assistant to conduct response surface correlation analysis, the RSM model regression equation was established based on the data in Table 3 and Formulas (1) and (2), and the following model was obtained:

(13) $$\begin{array}{ll} & F_{y}=5.665+0.242\times a_{p}-0.0029\times v+0.0255\times f+0.00004\times a_{p}\times v\\ & +0.0014\times a_{p}\times f-0.000036\times v\times f-0.0024\times a_{p}^{2}+0.0000023\times v^{2}\\ & -0.000195\times f^{2} \end{array}$$

(14) $$\begin{array}{ll} & A_{y}=1.5432-0.0059\times a_{p}-0.0006\times v-0.0187\times f-0.000004\times a_{p}\times v\\ & -0.00005\times a_{p}\times f+0.000009\times v\times f+0.0000000005\times v^{2}\\ & -0.000011\times f^{2}+0.000271\times a_{p}^{2} \end{array}$$

The response function can be geometrically interpreted by its corresponding response surface. This surface visually represents the response or how a dependent factor varies with an independent element. Response surface analysis is performed using the fitted approximate surface. If the fitted surface is a reliable approximation of the true response function, then analyzing the fitted surface is roughly equivalent to analyzing the actual process. The adequacy of the fitted model is generally assessed via the analysis of the variance of the residuals and the coefficient of determination $R^{2}$.

This study assessed the reliability of the model from both statistical and experimental perspectives via variance analysis and experimental verification. The ANOVA results for $F_{y}$ and $A_{y}$ are listed in Table 4 and Table 5, respectively.

Table 4 indicates that the F-value of the $F_{y}$ model is 106.97, demonstrating that the established $F_{y}$ model is significant, and there is only a 0.01% chance that such a large F-value is due to noise. The model terms v, $a_{p}$, and $v^{2}$ are significant, as a *p*-value less than 0.05 indicates. The $R^{2}$ value and adjusted-$R^{2}$ value of the $F_{y}$ model are 97.29% and 96.2%, respectively, and the difference between the two is negligible, indicating that the established polynomial can fully reflect the relationship between the design variables and the response.

Table 5 reveals that the F-value of the $A_{y}$ model is 289.12, indicating that the established $A_{y}$ model is significant, and there is only a 0.01% probability that such a large F-value is due to noise. The model terms v, f, $v\times a_{p}$, v×f, and $a_{p}^{2}$ are significant, as a *p*-value less than 0.05 indicates. The $R^{2}$ value and adjusted-$R^{2}$ value of the $A_{y}$ model are 99.30% and 99.39%, respectively, and the difference between the two is negligible, demonstrating that the established polynomial can fully reflect the relationship between the design variable and the response. Based on the ANOVA results, all of the constructed regression models can be used to quantify the relationship between process factors and corresponding responses.

Apart from the statistical analysis, validation experiments were conducted to verify the reliability of the constructed model. The verification test parameters were randomly selected within the experimental interval and differed from those in Table 6. Subsequently, the experimentally measured $F_{y}$ and $A_{y}$ values were compared with the calculated results of the established model. The relative errors between the experimental and predicted results are presented in Table 6. Based on the table, the maximum relative errors of $F_{y}$ and $A_{y}$ were found to be 7.63% and 7.01%, respectively. In line with existing research, these errors fall within the acceptable range, indicating that the model can be utilized.

**Figure 3:**
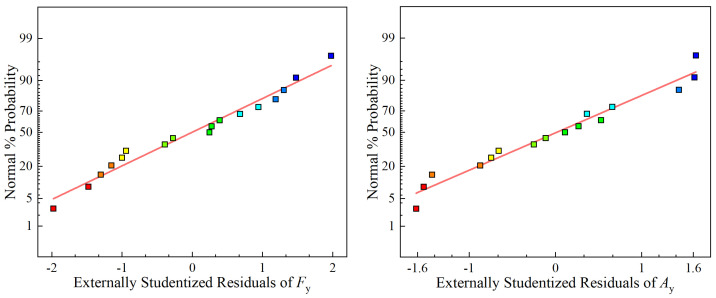
Normal plot of $F_{y}$ and $A_{y}$.

**Figure 4:**
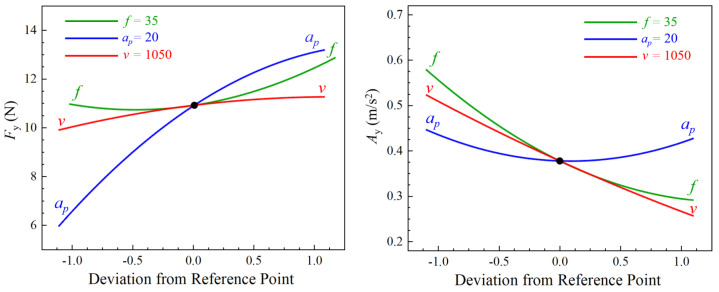
Perturbation plots of $F_{y}$ and $A_{y}$.

Figure 3 displays the normal probability plot of the $F_{y}$ and $A_{y}$ residuals. The plot indicates that the majority of the residuals are closely clustered around the straight reference line, signifying a normal distribution. This suggests that both regression models are well fitted. Figure 4 depicts a perturbation plot that shows the effect of the three factors on $F_{y}$ and $A_{y}$. This plot is a valuable graphical tool for comparing the effects of all of the factors at a particular point in the design space. The results indicate that the cutting depth significantly influences the cutting force, whereas the feed rate has the least effect. In terms of vibration, both cutting speed and feed rate have the most significant influence, while the cutting depth has the least effect.

**Figure 5:**
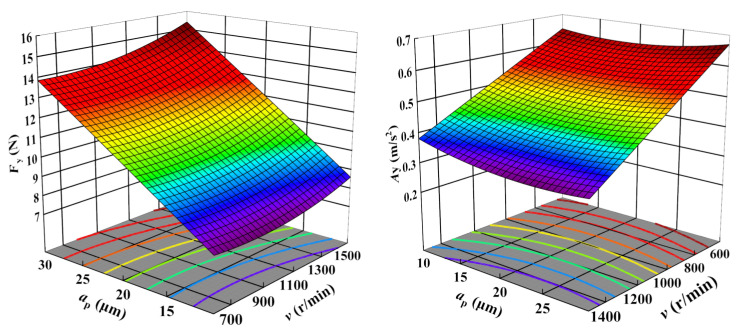
Influence of depth of cut and cutting speed on $F_{y}$ and $A_{y}$.

**Figure 6:**
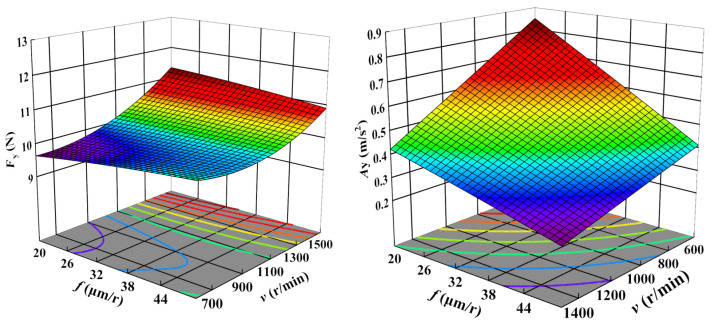
Influence of feed rate and cutting speed on $F_{z}$ and $A_{z}$.

**Figure 7:**
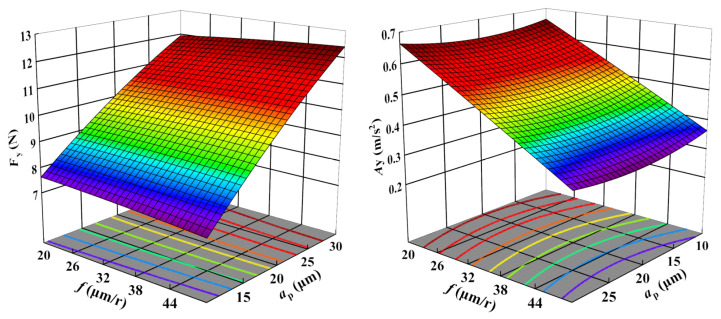
Influence of feed rate and depth of cut on $F_{z}$ and $A_{z}$.

Figure 5, Figure 6 and Figure 7 display the 3D contour plots illustrating the impact of cutting parameters on cutting force and vibration. Each plot represents the impact of two process variables, with all variables taking values within the experimental study range, while the other variable is fixed at the center point value. The experimental results demonstrate that the cutting force is most significantly affected by the depth of cut, followed by the cutting speed, while the feed rate has the least effect. As the depth of cut and cutting speed decrease, the cutting force also decreases. Concerning vibration, the cutting speed is the parameter with the most significant impact, followed by the feed rate, while the depth of cut has the least influence. The vibration tends to decrease with increased cutting speed and feed rate.

### 3.2. Optimization of Turning Parameters Based on NSDBO

**Figure 8:**
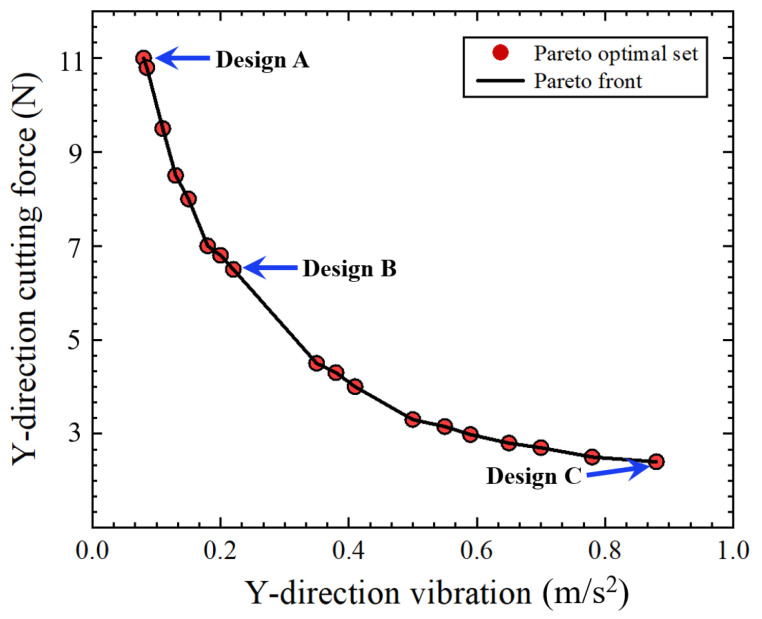
Pareto front of optimal process outputs.

The goal of the NSDOB optimization algorithm is to minimize the *y*-direction force and vibration during the cutting process of WHAs. The 1 and −1 levels of the experimental input parameters cutting speed, feed rate, and depth of cut are used as the initial population generation range of the genetic algorithm. The optimization algorithm uses a population size of 18, and the ratio of the ball-rolling dung beetle, the brood ball, the small dung beetle, and the thief beetle in the population is 1:1:1:1. The maximum number of iterations is set to 200 to ensure that the algorithm runs to completion. The optimized Pareto front obtained after 87 algorithm iterations is shown in Figure 8.

After obtaining the Pareto front, we obtained an optimal solution set containing 18 solutions. The solution close to Design A can obtain a smaller vibration, but larger cutting force. The solution close to Design B can obtain a smaller cutting force, but larger vibration. Therefore, after comprehensive consideration, we chose the solution with moderate vibration and force as the optimal solution. Table 7 illustrates the comparison between the optimization results of the algorithm and the optimal parameters obtained in the experiment. The cutting force obtained via the optimized parameters is reduced by 9.7%, and the vibration is reduced by 46.47%. The surface roughness of the final workpiece is reduced by 18.2%.

**Figure 9:**
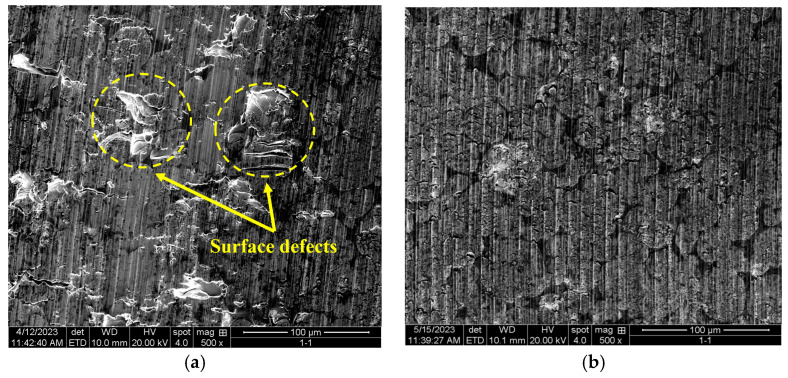
Comparison of surface topography of processed workpieces: (**a**) processing result before parameter optimization ($v=1050\;r/\min,\;a_{p}=30\;\mu m,\;f=20\;\mu m/r$); (**b**) processing result after parameter optimization ($v=1452\;r/\min,\;a_{p}=20.5\;\mu m,\;f=31.25\;\mu m/r$).

In order to verify the feasibility of the NSDBO algorithm, the turning experiment was carried out again using the optimized parameters, and the experimental conditions were the same as the orthogonal experimental conditions mentioned above. After the test, the surface morphology of the workpiece was observed using a scanning electron microscope. Figure 9 is a comparison of the surface morphology before and after optimization. The surface processed using the parameters before optimization is shown in Figure 9a, and the surface damage caused by incomplete material removal can be clearly seen. The surface processed with the optimized parameters is shown in Figure 9b. In contrast, regular knife lines were obtained without obvious damage on the surface, and the surface quality significantly improved.

In order to verify the superiority of the proposed algorithm, this method was compared with the genetic algorithm and the cuckoo algorithm, and the comparison results are shown in Table 8. It can be seen that the multi-objective dung beetle algorithm has a faster convergence speed and better global optimization performance.

## 4. Conclusions

In this paper, based on the dung beetle optimization algorithm, non-dominated sorting technology was introduced, and a multi-objective dung beetle optimization algorithm was proposed and combined with the RSM method for the optimization of the turning parameters of tungsten alloys. The main contributions of the article can be drawn as below:

- A second-order polynomial regression model using response surface methodology (RSM) was established to correlate cutting speed, feed rate, cutting depth, cutting force, and vibration. ANOVA analysis results indicated that all of the constructed regression models could be used to quantify the relationship between cutting parameters and corresponding responses.
- The influence of cutting parameters on cutting force and vibration was analyzed via RSM. The influence of depth of cut on cutting forces is the most significant, the influence of cutting speed is second, and the influence of feed rate is the smallest. For vibration, the cutting parameter with the most significant impact is the cutting speed, followed by the feed rate, and the depth of cut has the least influence.
- A multi-objective dung beetle optimization algorithm was realized by introducing the non-dominated sorting technology. The results show that the proposed algorithm has a faster convergence speed and better global optimization ability than the multi-objective genetic and cuckoo algorithms.
- Combining the RSM and NSDBO methods, the cutting parameters of the tungsten alloy were optimized. After the optimized parameters, the cutting force was reduced by 9.7%, and the vibration was reduced by 46.47%. The surface roughness of the final workpiece was reduced by 18.2%. This means that the method proposed in this text can guide the turning of tungsten alloys.

## Figures and Tables

**Table 1:** Chemical element composition and physical properties of 95W-3.5Ni-1.5Fe.

**Properties**	**Values**	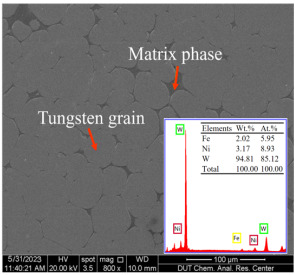
Density (g/cm^3^)	18.3	
Hardness (HV)	380	
Tensile strength (Mpa)	800	
Yield strength (Mpa)	400	
Young’s modulus (Gpa)	450	
Deflective strength (Mpa)	1176	
Thermal conductivity (W/(m·K))	89.2	
Thermal expansion coefficient (ppm/k)	4.8	

**Table 2:** Levels of cutting parameters.

Symbol	Factors	Unit	Level		
			−1	0	1
v	Cutting speed	r/min	800	1100	1400
f	Feed rate	μm/r	20	35	50
ap	Depth of cut	μm	2	4	6

**Table 3:** Experimental layout and measurement results.

Exp. No.	*v* (r/min)	*a*_p_ (μm)	*f* (μm/r)	*F*_y_ (N)	*A*_y_ (m/s^2^)	*R*_a_ (nm)
1	600	30	35	11.79	0.662	461
2	600	20	20	9.588	0.851	557
3	600	10	35	7.716	0.653	454
4	600	20	50	10.15	0.415	332
5	1050	30	50	12.57	0.343	289
6	1050	20	35	9.904	0.488	321
7	1050	30	20	12.10	0.672	411
8	1050	10	20	7.492	0.656	438
9	1050	10	50	7.128	0.355	193
10	1050	20	35	9.879	0.498	346
11	1050	20	35	10.25	0.485	355
12	1050	20	35	10.16	0.472	368
13	1050	20	35	10.34	0.488	329
14	1500	30	35	13.29	0.332	398
15	1500	10	35	8.496	0.389	409
16	1500	20	50	10.97	0.236	206
17	1500	20	20	11.37	0.416	478

**Table 4:** ANOVA for cutting $F_{y}$.

Source	Sum of Squares	Degrees of Freedom	Mean Square	F-Value	*p*-Value	
Model	43.34	9	5.48	106.97	<0.0001	Significant
v	2.98	1	2.98	58.13	0.0001	
$a_{p}$	44.74	1	44.74	872.95	<0.0001	
f	0.009	1	0.009	0.1752	0.6881	
$v\times a_{p}$	0.1296	1	0.1296	2.53	0.1558	
v×f	0.2314	1	0.2341	4.51	0.0712	
$a_{p}\times f$	0.1739	1	0.1739	3.39	0.1080	
$v^{2}$	0.8782	1	0.8782	17.14	0.0044	
$a_{p}^{2}$	0.2431	1	0.2431	4.74	0.0658	
$f^{2}$	0.0081	1	0.0081	0.1576	0.7032	
Residual	0.3587	7	0.0512			
Cor total	40.70	16				
						
Standard deviation		0.2264		$R^{2}$		0.9729
Mean		10.19		Adjusted $R^{2}$		0.9620
Coefficient of variation		2.22		Predicted $R^{2}$		0.9341
Predicted residual of sum of squares		3.27		Adequate precision		35.6818

**Table 5:** ANOVA for cutting $A_{y}$.

Source	Sum of Squares	Degrees of Freedom	Mean Square	F-Value	*p*-Value	
Model	0.3975	9	0.0442	289.12	<0.0001	Significant
v	0.1824	1	0.1824	1194.1	<0.0001	
$a_{p}$	0.0002	1	0.0002	1.58	0.2485	
f	0.1914	1	0.1914	1270.41	<0.0001	
$v\times a_{p}$	0.0011	1	0.0011	7.13	0.0320	
v×f	0.0164	1	0.0164	107.26	<0.0001	
$a_{p}\times f$	0.0002	1	0.0002	1.28	0.2946	
$v^{2}$	0.0001	1	0.0001	0.0003	0.9872	
$a_{p}^{2}$	0.0031	1	0.0031	20.24	0.0028	
$f^{2}$	0	1	0	0.1588	0.7022	
Residual	0.0011	7	0.0002			
Cor total	0.3986	16				
						
Standard deviation		0.0124		$R^{2}$		0.9930
Mean		0.4935		Adjusted $R^{2}$		0.9939
Coefficient of variation		2.5		Predicted $R^{2}$		0.9809
Predicted residual of sum of squares		0.0076		Adequate precision		64.7200

**Table 6:** Results of confirmation experiments and their comparison with predicted values.

Exp. No.	Design Parameters			$F_{y}\;(N)$			$A_{y}\;(m/s^{2})$		
	v (r/min)	$a_{p}$ (μm)	f (μm/r)	Exp.	Predicted	Error (%)	Exp.	Predicted	Error (%)
1	600	35	30	11.735	12.48	6.34%	0.839	0.7863	6.28%
2	600	20	20	9.432	9.630	2.09%	0.892	0.8500	4.70%
3	1050	50	30	13.75	14.80	7.63%	0.717	0.7672	7.01%
4	1050	35	20	13.15	12.71	3.34%	0.657	0.6981	6.25%

**Table 7:** Comparison of cutting parameters.

Parameters	*v* (r/min)	*a*_p_ (μm)	*f* (μm/r)	$F_{y}\;(N)$	$A_{y}\;(m/s^{2})$
Design A	1032	25.85	22.85	13.87	0.0921
Design B	1452	20.5	31.25	6.43	0.19
Design C	897	12.8	21.52	5.62	0.92
Experimental	1050	30	20	7.128	0.355

**Table 8:** Comparison results with similar algorithms.

Method	Maximum Iterations	Optimized Result	
		$F_{y}\;(N)$	$A_{y}\;(m/s^{2})$
NSDBO	87	6.43	0.19
NAGA-II	112	8.92	0.27
CSO	95	9.1	0.21

## Data Availability

Not applicable.
